# Primary pericardial mesothelioma complicated by pericardial calcification

**DOI:** 10.1186/s12872-023-03142-w

**Published:** 2023-03-08

**Authors:** Jian Zhang, Daxing Liu, Dengshen Zhang, Ke Guo, Xiaorong Yang

**Affiliations:** Department of Cardiac Macrovascular Surgery, Affiliated Hospital of Zunyi Medical University, No. 149, Dalian Road, Huichuan District, Zunyi City, 563003 Guizhou Province China

**Keywords:** Case report, Misdiagnosis, Computed tomography, Pericardial calcification, Primary pericardial mesothelioma

## Abstract

**Background:**

Pericardial calcification is usually a marker of chronic diseases, and its occurrence in rapidly progressing malignant primary pericardial mesothelioma (PPM) is extremely rare. Therefore, this atypical imaging appearance contributes to more frequent misdiagnosis of PPM. However, no systematic summary currently exists of the imaging characteristics of malignant pericardial calcification in PPM. In our report, its clinical characteristics are discussed in detail, to provide a reference to reduce the misdiagnosis rates of PPM.

**Case presentation:**

A 50-year-old female patient was admitted to our hospital, presenting primarily with features suggestive of cardiac insufficiency. Chest computed tomography revealed significant pericardial thickening and localized calcification, suspicious of constrictive pericarditis. A chest examination performed through a midline incision showed a chronically inflamed and easily-ruptured pericardium that was closely adherent to the myocardium. Post-operative pathological examination confirmed a diagnosis of primary pericardial mesothelioma. Six weeks postoperatively, the patient experienced symptom recurrence and abandoned chemotherapy and radiation therapy. Nine months postoperatively, the patient died of heart failure.

**Conclusion:**

We report this case to highlight the rare finding of pericardial calcification in patients with primary pericardial mesothelioma. This case illustrated that confirmation of pericardial calcification cannot completely rule out rapidly developing PPM. Therefore, understanding the different radiological features of PPM can help to reduce its rate of early misdiagnosis.

## Background

Primary pericardial mesothelioma (PPM) is a rare and highly aggressive type of pericardial tumor. A large number of previous autopsies showed that PPM has a prevalence rate of less than 0.0022% and a median survival of 2–6 months [1]. PPM presents with varying degrees of cardiac insufficiency due to the variability of restrictions imposed by pericardial effusion or pericardial masses on the motion of the heart. On imaging, PPM often manifests as pericardial effusion or pericardial thickening. PPM complicated by pericardial calcification is an extremely rare condition, and only one such case has been reported by the European Heart Journal to date (in 2020) [2]. Hence, PPM may have rare imaging manifestations, such as pericardial calcification, which can easily cause its misdiagnosis or delayed diagnosis [3].

## Case presentation

A 50-year-old female patient developed shortness of breath, fatigue, and edema of both lower extremities 3 weeks before presentation. Two weeks after the onset of symptoms, she visited a local hospital and underwent transthoracic color Doppler echocardiography, which revealed a large pericardial effusion. She also underwent ultrasound-guided pericardiocentesis, and > 1200 mL of dark red pericardial fluid was aspirated. She received oral diuretics, including furosemide, and spironolactone, with no significant relief of symptoms.

The patient was referred to our hospital 1 week later. Physical examination revealed a body temperature of 36.6 °C, heart rate of 112 beats per minute, regular heart rhythm, respiration rate of 32 breaths per minute, distal blood oxygen saturation of 91%, blood pressure of 124/70 mmHg, jugular vein distention, decreased breath sounds over the lower lung lobes bilaterally, distant heart sounds, mild edema of both lower extremities, and venous pressure (measured at the elbow) of 41 cmH_2_O.

**Fig. 1:**
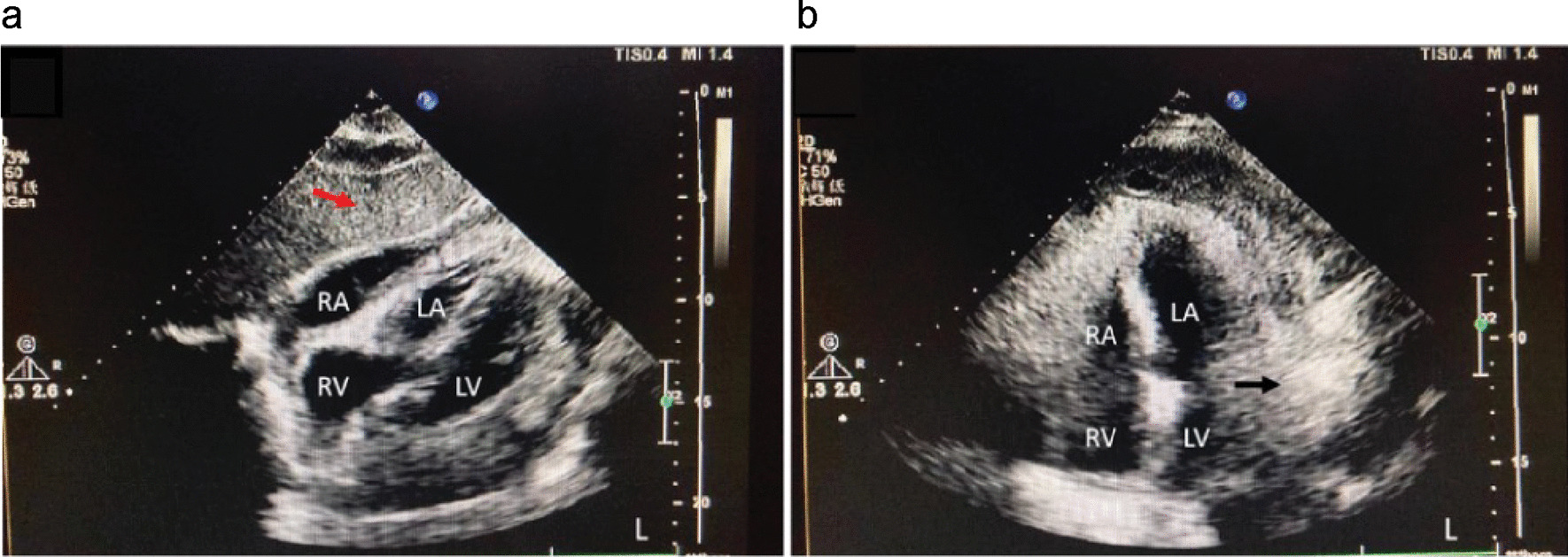
Transthoracic echocardiography. Transthoracic echocardiograph revealing thickening of the anterior pericardial wall (**A** red arrow) and uneven hyperechoic masses extending to the pericardial cavity from the pericardium along the lateral wall of the left ventricle (**B** black arrow)

No significant hematological or biochemical abnormalities were found (an albumin level of 29 g/L, slightly lower than the normal of 35 g/L); the carbohydrate antigen level was 125: 863 U/mL; test results were negative for other markers, including alpha-fetoprotein, carcinoembryonic antigen, ferritin, and human chorionic gonadotropin. Electrocardiography tracing suggested a sinus rhythm and low voltage in the limb leads. Transthoracic color Doppler echocardiography revealed a 4-mm thickening of the anterior pericardial wall, a 6-mm thickening of the posterior pericardial wall, a small pericardial effusion in a 64 × 14-mm uneven hyperechoic area along the lateral wall of the left ventricle (Fig. 1), mild mitral valve regurgitation, mild tricuspid valve regurgitation, normal left ventricular systolic function (left ventricular ejection fraction, 53%; left ventricular fractional shortening (FS), 31%), left ventricular diastolic function E/A > 1, and diagram of mitral annular velocity measured using Doppler Tissue Imaging (TDI): e: 11 cm/s, a: 8 cm/s, e/a > 1.

**Fig. 2:**
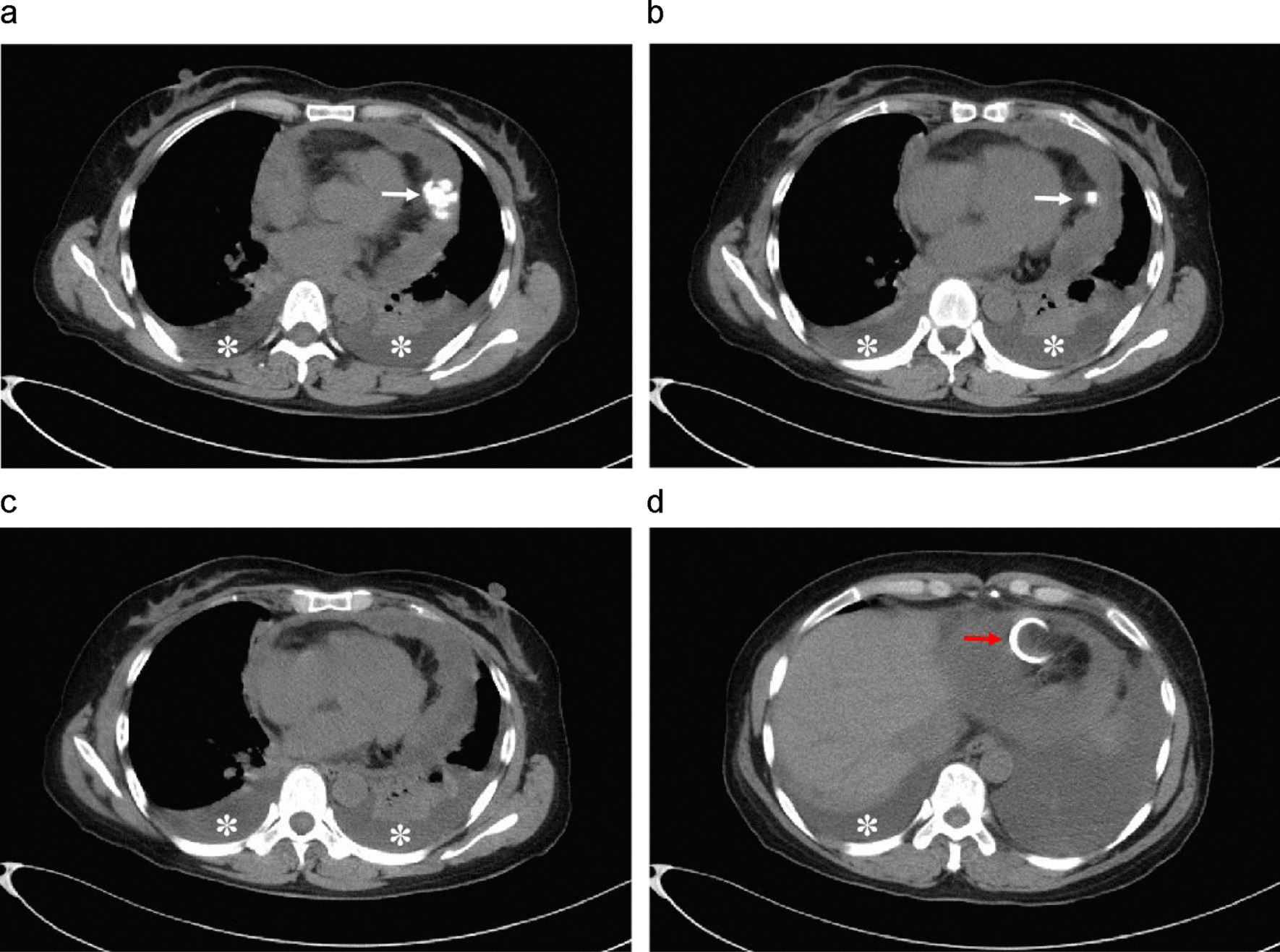
Chest computed tomography (CT). Chest CT scans (soft-tissue window, axial plane) revealing diffuse thickening of the pericardium, more prominent along the left lateral wall with notable calcified foci (**A**, B white arrow), bilateral pleural effusion (**B**, **C** stellate pattern), and an 8 F “pigtail” perforated pericardial drainage catheter (**D** red arrow)

**Fig. 3:**
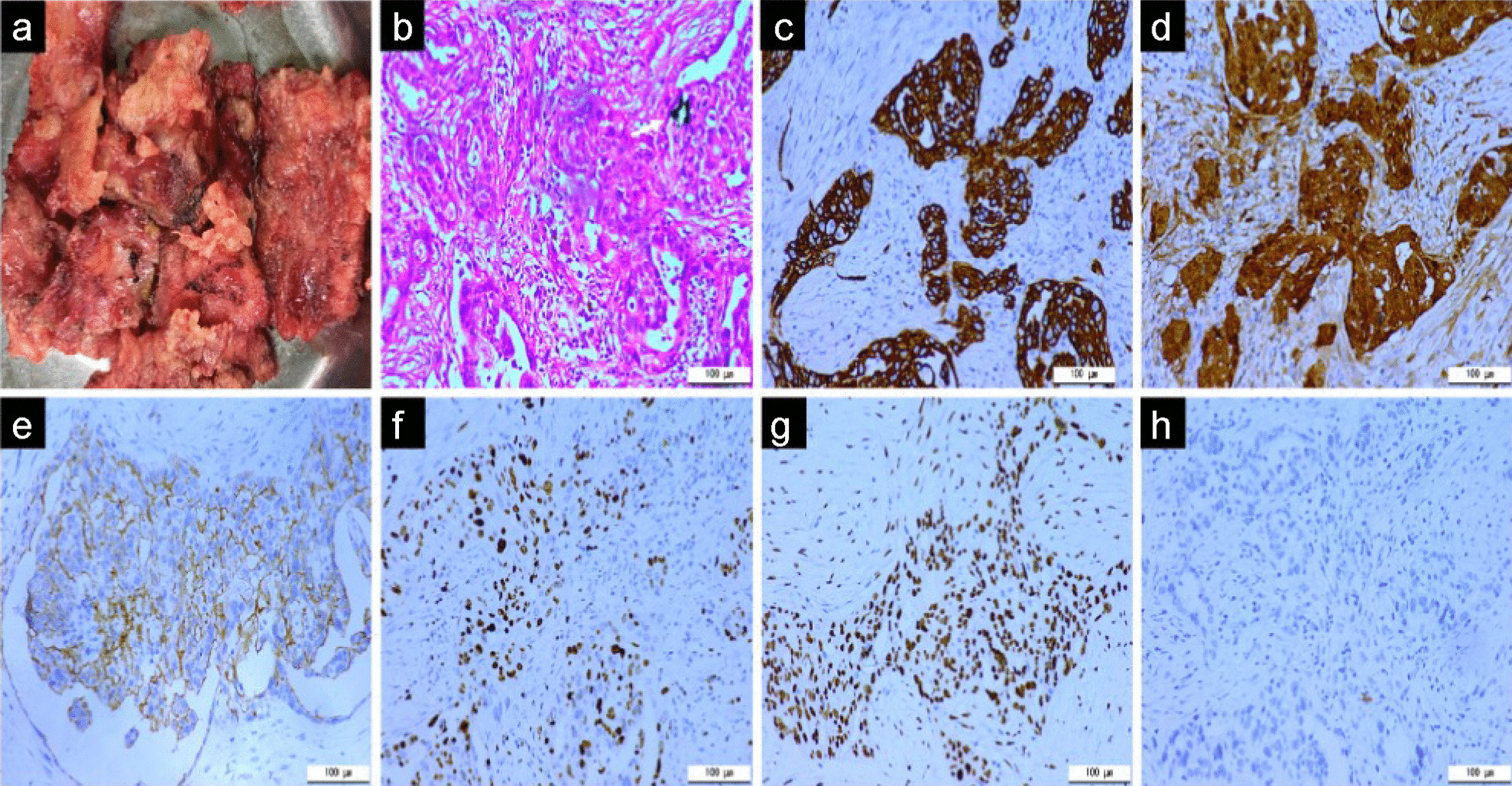
Histological and immunohistochemical analyses. Histological and immunohistochemical analyses of the specimens: brittle and greyish white pericardial masses with rich blood supply (**A**); papillary and glandular clusters of epithelioid tumor cells with eosinophilic cytoplasm and calcified foci (white arrow) (**B**) HE×200; positive immunohistochemical reaction for CK protein (**C**) IHC×200; positive immunohistochemical reaction for calretinin protein (**D**) IHC×200; positive immunohistochemical reaction for D2-40 protein (**E**) IHC×200; 60% hotspots as indicated by the Ki67 proliferative index (**F**) IHC×200; positive immunohistochemical reaction for WT-1 protein (**G**) IHC×200; and negative immunohistochemical reaction for BerEP4, EMA, napsin-A, and TTF-1(**H**) IHC×200.

Chest computed tomography (CT) examinations suggested prominent thickening of the pericardium (25 mm), uneven thickening of the left and right lateral walls, multiple pericardial calcifications along the left lateral wall (Fig. 2), and a small amount of bilateral pleural effusion. The patient had no history of asbestos exposure or tuberculosis. Since a preliminary diagnosis of tuberculous constrictive pericarditis was considered, we performed a chest exploration and partial pericardiectomy through a midline incision. Post-operative findings suggested brittle and grayish-white pericardial masses with rich blood supply (Fig. 3A) and pericardial calcification located in the left pericardium adjacent to the phrenic nerve along the left ventricle and embedded in the myocardium. After partial pericardiectomy, the patient’s central venous pressure dropped from 26 cm H_2_O to 7 cm H_2_O. The symptoms of fatigue and shortness of breath improved after surgery. Hematoxylin and eosin staining revealed papillary, micropapillary, and glandular clusters of epithelioid tumor cells with eosinophilic cytoplasm and vesicular nuclei, focal calcifications, and few mitotic figures. Immunohistochemistry (IHC) results revealed the following: cytokeratin (CK) (++); calretinin (++); CK5/6 (++); WT-1 (++); D2-40 (+); BerEP4 (−); EMA (−); napsin-A (−); thyroid transcription factor-1 (−); and Ki67 (hotspots 60%) (Fig. 3B–H). The final pathological diagnosis was confirmed to be primary malignant epithelioid pericardial mesothelioma. After surgery, the patient continued to receive oral diuretics (torsemide 20 mg/day and spironolactone 25 mg/day). Six weeks after surgery, the patient experienced re-aggravation of symptoms of cardiac insufficiency and abandoned chemotherapy and radiation therapy. Nine months after the surgery, the patient died of heart failure.

## Discussion and conclusions

PPM usually manifests as pericardial thickening on chest CT images, and patients with PPM can also present with localized masses and cysts combined with a large amount of pericardial and pleural effusion [2, 4]. However, PPM presenting with pericardial calcification is extremely rare. Pericardial calcification usually occurs secondary to inflammatory or traumatic diseases and is characterized by the formation of calcified fibrous pericardial adhesions between the pericardium and epicardium [5]. Pericardial calcification usually serves as a marker of chronic diseases [6]; however, PPM is a malignant tumor originating in the serous pericardium with an extremely poor prognosis and a median survival time of ≤ 6 months from diagnosis [1, 3]. Therefore, pericardial calcification secondary to PPM is extremely rare. To date, only one such case has been reported in the literature [2]. The patient in this case report underwent pericardial needle biopsy 2 months before referral to our hospital and had a history of chronic iatrogenic hematoma, which is considered to be a possible cause of pericardial calcification. However, pericardial hematoma simulated by the injection of blood into the pericardium subsided without residual calcifications in previous in vivo animal studies [7]. Calcified hematoma mainly occurs after high-energy trauma and induces pericardial calcification by altering fibrinolysis and fibrin absorption. Although this patient had a history of pericardial needle biopsy, pericardial calcification was identified on chest CT re-examination 2 weeks after the biopsy. Absorption and calcification of pericardial hematoma in such a short time are not entirely justified. Moreover, calcified lesions were located in the left pericardium adjacent to the left phrenic nerve, which is relatively distal to the biopsy site. Hence, the pericardial needle biopsy was excluded as a possible cause of pericardial calcification in this patient. Similarly, the author of the previous case report believes that the cause of PPM combined with pericardial calcification is associated with the pathological characteristics of PPM. The patient reported by this author had PPM that was pathologically classified as sarcomatoid PPM. In terms of histopathological types, malignant osteoid components were also found in three reported cases of sarcomatoid and biphasic pleural mesothelioma, which manifested as calcified pleural masses on imaging findings [8]. The patient in our case report had epithelioid pericardial mesothelioma according to pathological classification, and there are currently no case reports of epithelioid pericardial mesothelioma combined with pericardial calcification. The formation of focal calcified lesions was still observed in hematoxylin and eosin-stained slices of the pericardium distal to the area of pericardial calcification in this patient; thus, epithelioid mesothelioma can still provide the pathological basis underlying the formation of calcified lesions given the diversity in the differentiation of mesothelial cells. In addition, the differentiation of pleural mesothelioma into osteosarcoma has already been reported in the literature [9, 10].

Pericardial calcification is commonly observed in patients with tuberculous constrictive pericarditis, radiation-induced pericarditis, or idiopathic pericarditis and those undergoing surgery [11]. Hence, PPM manifesting as pericardial calcification in these patients can easily be mistaken for tuberculous constrictive pericarditis (especially when the patient originates from regions with a high incidence of tuberculosis). In addition to pericardial calcification, there are other CT imaging features in this case report that are still similar to those in previous case reports on PPM. These include diffuse thickening of the pericardium, single or multiple nodules or mass shadows of different sizes observed within the pericardium, prominently thickened localized masses that can even compress the heart and lead to cardiac changes, and pericardial effusion or pleural effusion (usually complicating PPM). Pericardial effusion in PPM is mostly hemorrhagic and particularly prominent in patients with diffuse thickening of the pericardium. In patients with PPM, the symptoms of epicardial involvement can not only be attributed to obstruction of blood flow and cardiac tamponade, but also to congestive heart failure caused by myocardial infiltration [12]. Given the nonspecific signs and symptoms of PPM and its diverse imaging manifestations, While we understand that pericardial mesothelioma can be challenging to diagnose, however, diagnosis can be confirmed via a biopsy and histological examination as in this case. To reduce the misdiagnosis rate of PPM, we can associate the characteristics of malignant pericardial calcification with the following factors based on previous literature reports and an analysis of patient characteristics in this case report: (1) clinical manifestations of restrictive heart failure with no obvious cause and progressive changes in disease conditions within several weeks; (2) a large amount of pericardial effusion with hemorrhagic changes and no alleviation after repeated needle aspiration; (3) diffuse and uneven thickening of the pericardium with even and localized mass shadows; (4) pericardial calcification in irregular clumps, multilobed patterns, and burr-like changes to the boundary of calcified lesions; and (5) brittle pericardium with a rich blood supply and no clear boundary with the myocardium can be observed during surgery. Although the above-mentioned characteristics cannot confirm the diagnosis of PPM, they can serve as warning signs of malignant pericardial tumors and reduce the misdiagnosis of PPM as other benign pericardial calcifications. At present, there is no effective treatment for PPM, and PPM treatment is still based on therapeutic recommendations for pleural and peritoneal mesothelioma. For rapidly progressing PPM, the difficulty in early diagnosis is still one of the main reasons for its poor prognosis. Therefore, the survival time of such patients can be effectively extended by improving the early detection of PPM, reducing misdiagnosis, and adopting personalized, multidisciplinary, and comprehensive treatment strategies in clinical practice [13].

In conclusion, highly heterogeneous PPM cells can also evolve to form calcified lesions. Therefore, although extremely rare, radiological evidence of pericardial calcification may already be present in some PPM cases. Understanding the radiological features of malignant pericardial calcifications, combined with the rapidly evolving natural history, can help narrow the differential diagnosis and enable timely diagnosis of PPM.

## Data Availability

All data generated or analysed during this study are included in this published article.

## Abbreviations

PPM: Pericardial mesothelioma
TDI: Doppler tissue imaging
CT: Computed tomography
IHC: Immunohistochemistry
CK: Cytokeratin (CK)
